# Sclerobanding in the treatment of second and third degree hemorrhoidal disease in high risk patients on antiplatelet/anticoagulant therapy without suspension: a pilot study

**DOI:** 10.3389/fsurg.2023.1290706

**Published:** 2023-11-06

**Authors:** Francesco Pata, Luigi M. Bracchitta, Bruno Nardo, Gaetano Gallo, Giancarlo D’Ambrosio, Salvatore Bracchitta

**Affiliations:** ^1^ Department of Pharmacy, Health and Nutritional Sciences, University of Calabria, Rende, Italy; ^2^ PhD Program in Arterial Hypertension and Vascular Biology, Sapienza University, Rome, Italy; ^3^ Primary Care Department, ATS Città Metropolitana, Milan, Italy; ^4^ Department of Surgery, Sapienza University of Rome, Rome, Italy; ^5^ Department of General Surgery, Surgical Specialties and Organ Transplantation, Sapienza University of Rome, Rome, Italy; ^6^ Coloproctology Centre, Clinica del Mediterraneo, Ragusa, Italy

**Keywords:** hemorrhoids, anticoagulant, antiplatelet, hemorrhoidal disease, sclerobanding, haemorrhoids, sclerotherapy, rubber band ligation

## Abstract

**Introduction:**

Around 20% of population in western countries is under anticoagulant treatment. However, there is paucity of evidence about the treatment of HD in patients under anticoagulant/antiplatelet therapy, although both suspension and continuation in the perioperative period may increase the risk of severe complications. The aim of this pilot study was to confirm the feasibility and safety of sclerobanding (Combined Rubber Band Ligation with 3% Polidocanol Foam Sclerotherapy), an office-based procedure, for the treatment of second-and third-degree HD in patients under anticoagulant/antiplatelet therapy without suspension.

**Materials and methods:**

Patients affected by second-third-degree haemorrhoids unresponsive to conservative treatment and under anticoagulant/antiplatelet were enrolled between November 2019 and October 2021. Postoperative complications, readmission, mortality and reintervention during the follow-up were evaluated.

**Results:**

Fifty-one patients were recruited, 23 female (45.1%) and 28 male (54.9%), with an average age of 65 years ± 11.4 SD (range 42–90). Twenty-seven patients (52.9%) had II-degree haemorrhoidal disease, and 24 (47.1%) had grade III-degree. The most frequently taken medications were dual antiplatelet therapy (51%) and new oral anticoagulants (NOACs) (21.6%). The mean follow-up was 23 months. No intraoperative complications were recorded. The rate of complications in the first postoperative month was 13.7%, represented by mild complications: 6 cases of moderate to severe pain and 1 case (2%) of thrombosis of a residual haemorrhoidal nodule, all regressing after conservative therapy. No severe complications were reported. Postoperative complications were not statistically significantly associated with the number of nodules treated (1, 2, or 3), the disease grade (2nd vs. 3rd) or the specific anticoagulant/antiplatelet regimen. During follow-up, 2 patients (4%) required a new procedure for recurrent bleeding: one an infrared photocoagulation as outpatient, and another a haemorrhoidectomy after 3 months. No cases of intraoperative or postoperative mortality occurred.

**Conclusions:**

Sclerobanding is a safe and effective technique in treating intermediate-grade haemorrhoidal disease in patients at high risk on anticoagulant/antiplatelet therapy. Sclerobanding is repeatable, usually does not require anaesthesia, and is cost-effective. Observational multicentre studies with a larger number of patients and controlled clinical trials will be needed to confirm these results.

## Introduction

Approximately 1/5 of the United States population is on anticoagulant treatment (1), with 52% of them being treated with acetylsalicylic acid in the 45–75 year age group (2). The most common indications for therapy include primary prevention of ischemic heart disease and stroke, aortic-coronary stent placement, and treatment of deep venous thrombosis (3). In the ROCKET AF trial, a randomized control trial (RCT) comparing Rivaroxaban with Warfarin for the prevention of stroke and embolism in Atrial Fibrillation, 4.8% of patients experienced gastrointestinal bleeding episodes, with 29% being of rectal origin (4).

Haemorrhoidal disease is one of the most common causes of lower gastrointestinal bleeding (5), often self-limiting, but in some cases leading to severe anaemia requiring hospitalization, operative treatment, and/or transfusions (6). Treating haemorrhoidal disease in patients on anticoagulants represents a therapeutic challenge: on one hand, continuation of anticoagulant therapy is associated with a higher risk of postoperative bleeding; on the other hand, suspension itself can lead to new cardiovascular, neurological or thrombotic events, due to a rebound hypercoagulability phenomenon (7–10). Suspending antiplatelet therapy in secondary prevention may result in a new coronary or cerebrovascular event in 4.1% and 4.9% of cases, respectively (11, 12).

The use of office-based techniques, not requiring anaesthesia and hospitalization, mitigates but does not eliminate this risk. Rubber band ligation (RBL), reported as the most effective ambulatory technique for the treatment of haemorrhoidal disease, may rarely be associated with severe late bleeding (13), often occurring between the 10th and 14th postoperative day, and this risk is increased in patients on anticoagulant therapy (14, 15).

However, there is a gap in the literature: the few published studies on anticoagulant and antiplatelet-treated patients for haemorrhoidal disease are mostly case reports, case-series or single-centre retrospective studies with selection biases that reduce their external validity. The failure to conduct larger studies, after the initial preliminary cases reported, raises doubts about the clinical relevance of some of them (16, 17). Furthermore, the growing cohort of patients on new oral anticoagulants (NOACs) has been underinvestigated so far, despite the evidence of a higher incidence of rectal bleeding compared to warfarin (4).

The aim of this study was to assess the feasibility and safety of a novel technique named Sclerobanding (Combined Rubber Band Ligation with 3% Polidocanol Foam Sclerotherapy) on high-risk patients, with II or III degree haemorrhoidal disease, under anticoagulant/antiplatelet treatment, without suspending such therapy in the perioperative period.

## Materials and methods

Between November 2019 and October 2021, 51 patients underwent Sclerobanding at the Coloproctology Centre of *Clinica del Mediterraneo* Hospital in Ragusa, Italy. Inclusion criteria were:

- Bleeding II or III degree haemorrhoidal disease unresponsive to conservative treatment;
- Anticoagulant or antiplatelet therapy;
- High surgical risk (ASA 4) and/or high risk of thrombotic complications in case of therapy suspension, as assessed by the referring specialist;
- Increased risk of morbidity/mortality in case of anaemia as consequence of the recurrent bleeding;

Exclusion criteria were age under 18 years, pregnancy and concomitant anorectal pathologies;

The study was conducted according with the IDEAL Stage 2a framework.

Perioperative demographic data and postoperative complications (first postoperative days) were recorded in a local database. Each patient consented to the procedure after a discussion of indications, risks, and rationale of the Sclerobanding and the signature of a consent form. The Goligher classification was used to stage the disease. All procedures were performed by the same colorectal surgeon. Patients were seen at one week, one month, three months, six months, one year, and then every six months. The follow-up assessment included an interview to evaluate symptoms potentially related to complications or a possible recurrence (anal bleeding), inspection of the anorectal region in the Sims position, and digital rectal examination. An anoscopy was performed starting at the 3-month follow-up visit.

The primary aim of the study was to evaluate the feasibility and safety of Sclerobanding. The primary outcomes included postoperative morbidity, measured as the incidence of intraoperative adverse events and the complication rate within the first month, as well as the 30-day readmission rate. Secondary outcomes comprised the rate of reintervention during follow-up and patient satisfaction. The secondary objective was to investigate potential differences in postoperative complications based on the disease grade (second vs. third degree), the number of nodules treated during the procedure, and the anticoagulant regimen.

Patient satisfaction with the procedure was assessed using a visual analogue scale (VAS, 0-10) during the final follow-up visit.

The Sclerobanding technique has been described in the literature in 2021 (18, 19). No antibiotics is administered before the procedure. The patient is placed in a lithotomy position, and each haemorrhoidal nodule to be treated is ligated at the base above the dentate line to reduce the risk of postoperative pain, using a rubber band applied with a dedicated ligator. Subsequently, 2 or 3 ml of 3% polidocanol foam (obtained by mixing 2 ml of 3% polidocanol oil with 8 ml of air through two silicone-free syringes, one of 5 ml and one of 10 ml, connected by a connector) are injected into the ligated nodule. After the procedure, the patient is monitored for one hour and discharged with a dedicated phone contact number in case of need. Anaesthesia is typically not required; however, in some cases, patients may request local anesthesia. Postoperatively, analgesics and stool softeners are prescribed to minimize discomfort, postoperative pain, and the risk of bleeding. Patients are advised to abstain from alcohol consumption and smoking for at least the first month following the procedure.

Data are presented as means ± 1 standard deviation if normally distributed, range, or percentage. Fisher's exact test (alpha 0.05) was used to compare the rate of complications between patient subgroups (second vs. third degree; 1 vs. 2 vs. 3 nodules treated; complications according with different anticoagulant/antiplatelet regimens). A *p*-value < 0.05 was considered statistically significant. Statistical analysis was performed using Microsoft® Excel® 2016 (Microsoft Corporation, Redmond, WA, USA), and the online calculators https://www.socscistatistics.com/tests/fisher/default2.aspx and http://vassarstats.net/fisher2×4.html for Fisher's exact test.

## Results

Fifty-one consecutive patients were recruited, 23 female (45.1%) and 28 male (54.9%), with an average age of 65 years ± 11.4 SD (range 42–90). Twenty-seven patients (52.9%) had II-degree haemorrhoidal disease, and 24 (47.1%) had grade III-degree. Table 1 presents clinical, perioperative, and follow-up data for the patients included in the study.

**Table 1:** Demographic and postoperative outcomes of the patients included in the study.

	*n* (%)
**Patients**	51
Men	28 (54.9%)
Women	23 (45.1%)
**Age**	65 ± 11.4 (42–90) years
**Goligher's classification**	
2° degree	27 (52.9%)
3° degree	24 (47.1%)
**Follow-up**	23 months (6–24)
**Intraoperative complications**	0
**Postoperative complications (first 30 postop. days)**	7 (13.7%)
Moderate-severe pain (up to 20 days)	6 (11.7%)
Thrombosis of residual hemorrhoidal nodule	1 (2%)
**Readmission for postoperative complications**	0
**Mortality**	0
**New operations for recurrence**	2 (4%)
Infrared photocoagulation	1 (2%)
Hemorroidectomy	1 (2%)

**Figure 1:**
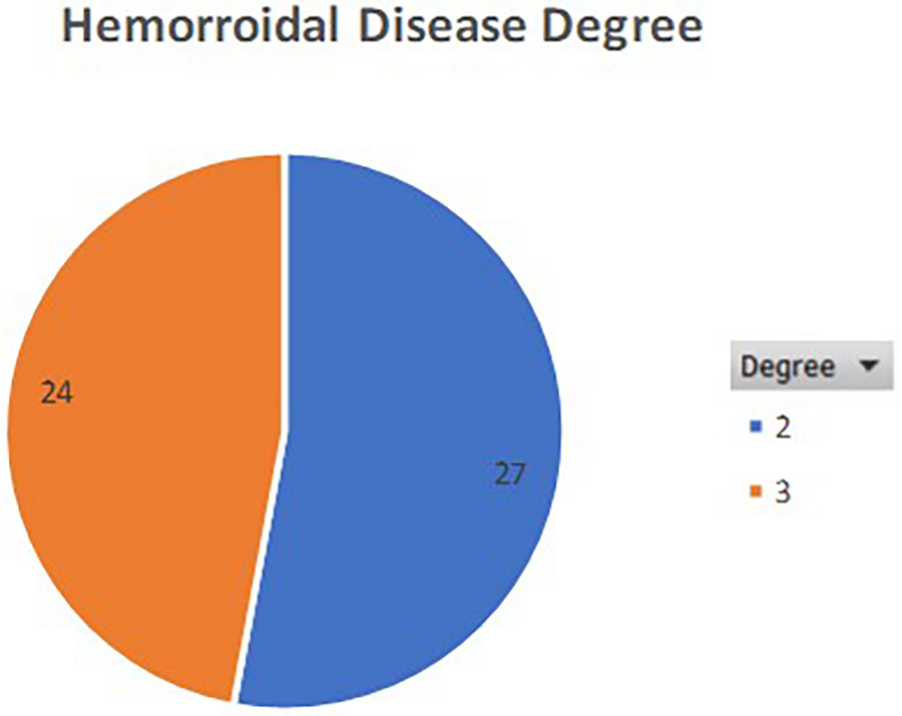
Grade of HD disease in the patients included in the study.

**Figure 2:**
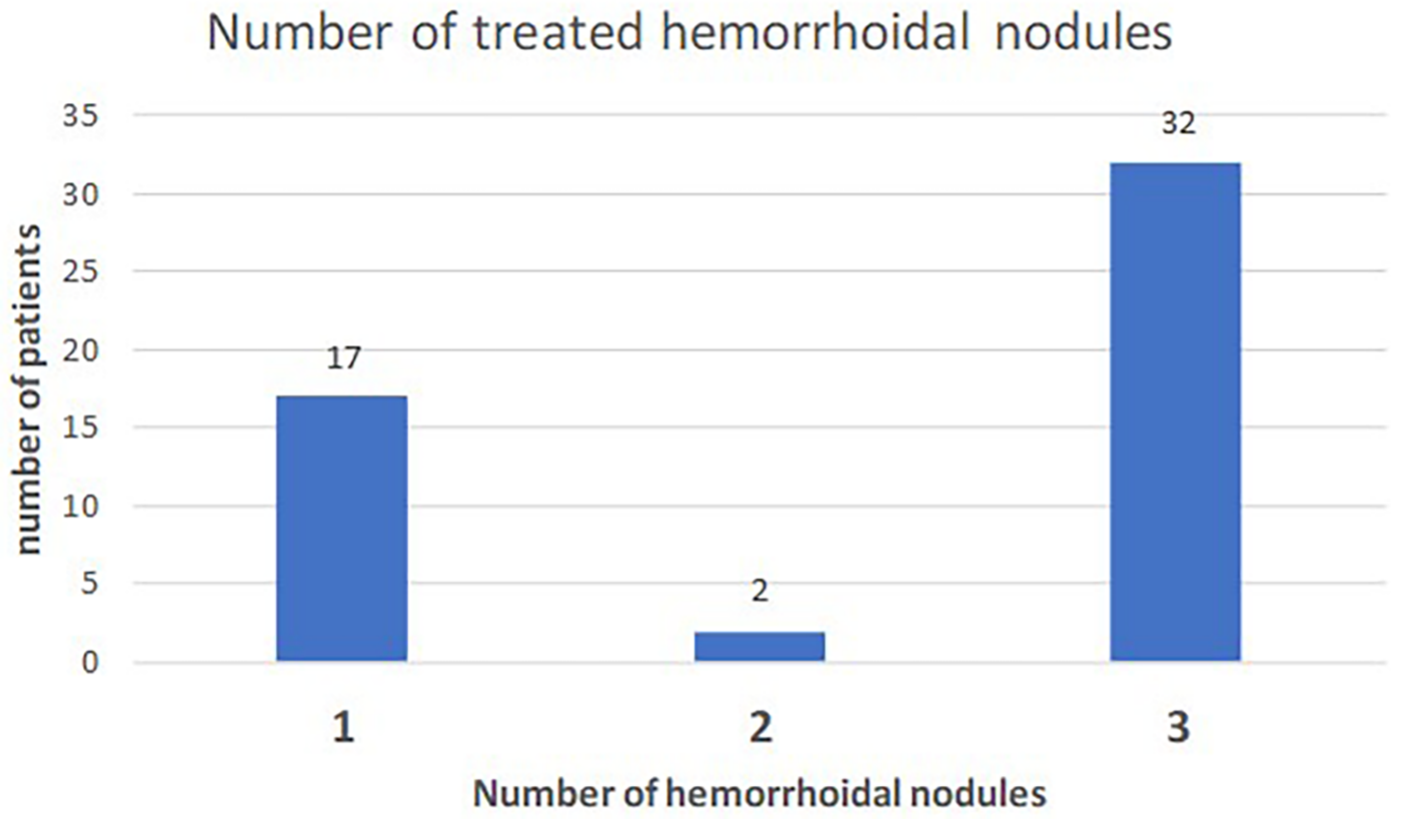
Number of hemorrrhoidal nodules treated per patient.

Figures 1, 2 show the degree of the disease and the number of nodules treated per patient.

**Figure 3:**
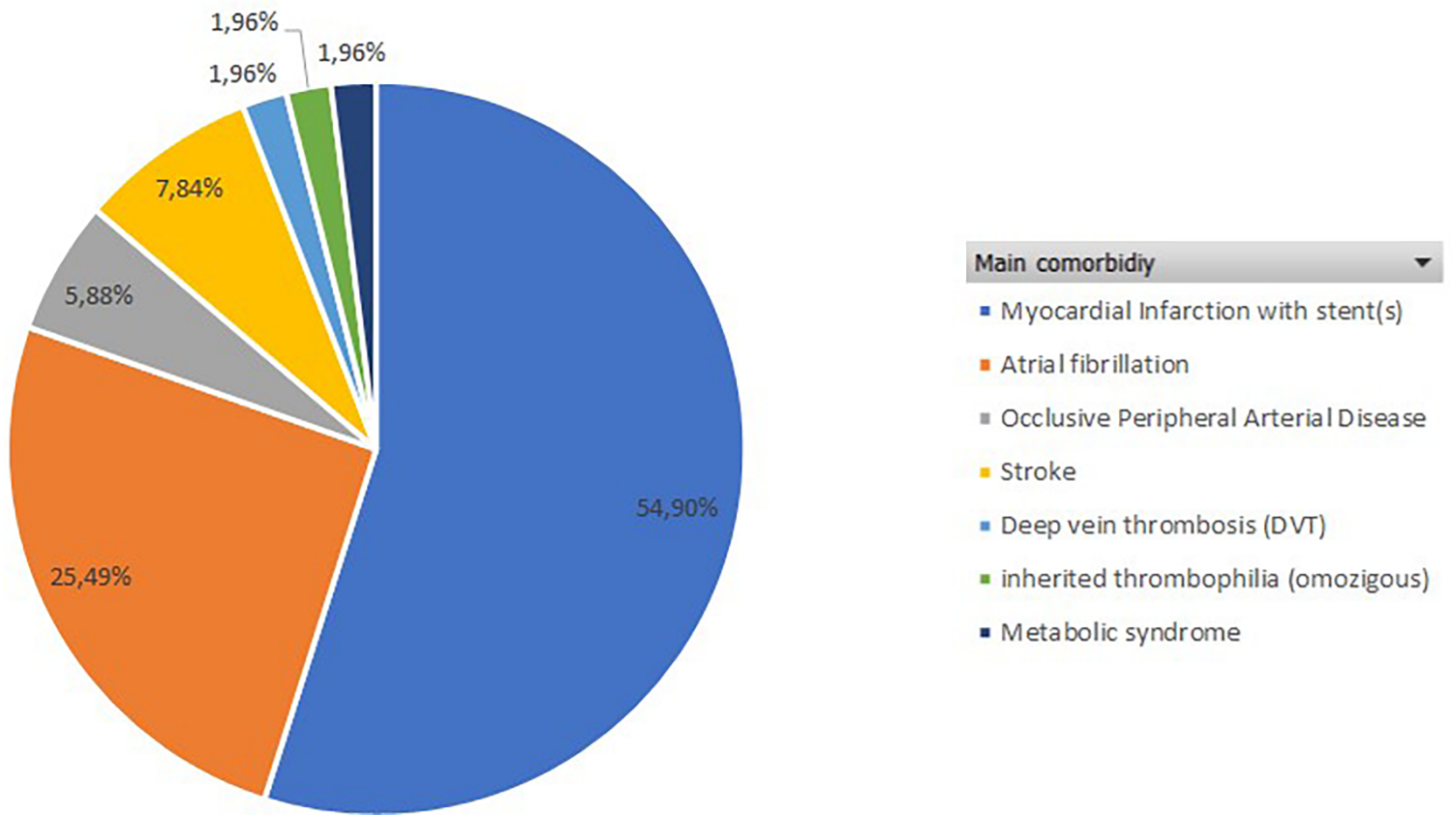
Main comorbidity in the patients recruited in the study.

**Figure 4:**
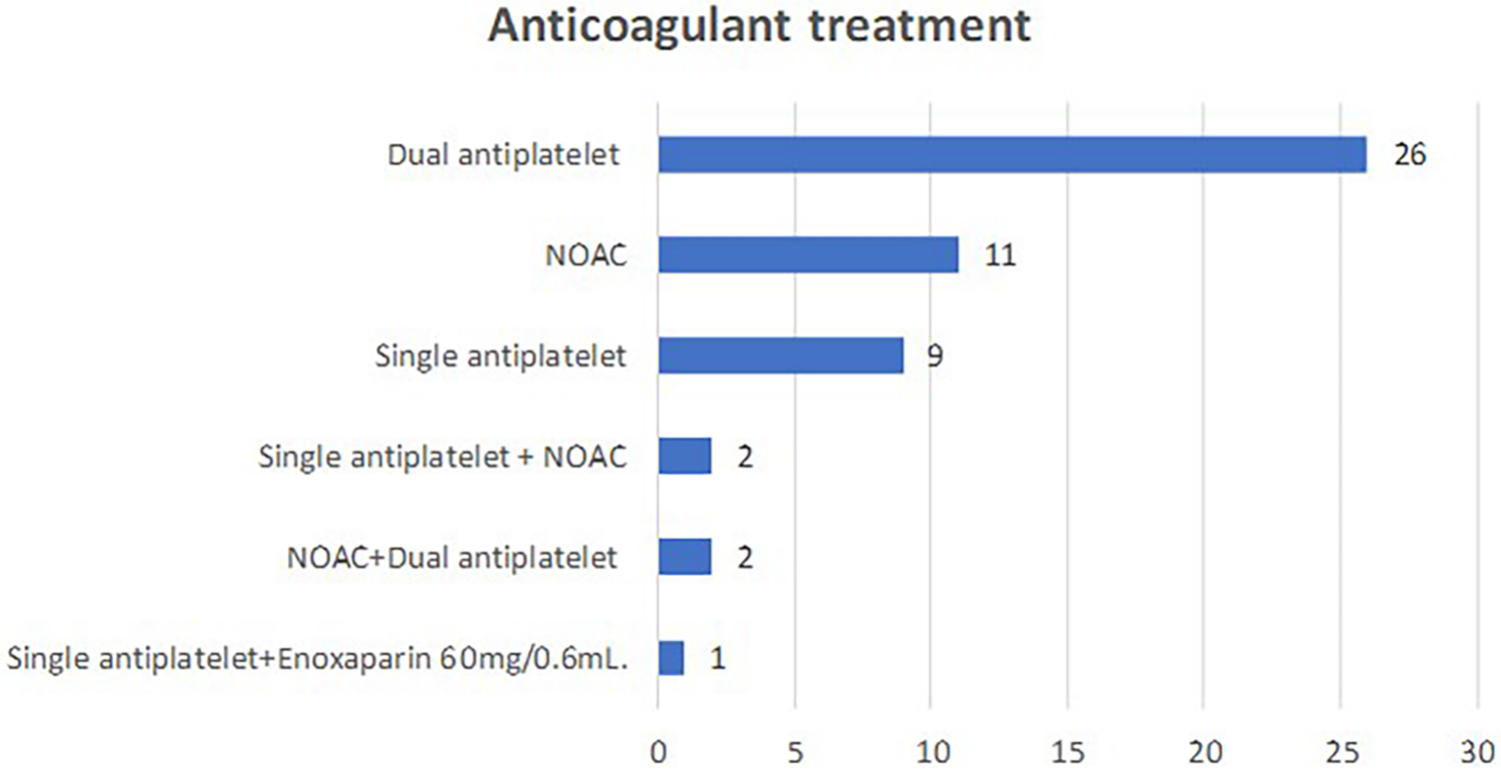
Anticoagulant regimen of the patients included in the study.

The most common comorbidities leading to anticoagulant therapy were ischemic heart disease with previous stent placement and atrial fibrillation (Figure 3), while the most frequently taken medications were dual antiplatelet therapy (51%) and new oral anticoagulants (NOACs) (21.6%), as illustrated in Figure 4.

The mean follow-up was 23 months. No intraoperative complications occured. The rate of complications in the first postoperative month was 13.7%, represented only by mild complications, particularly 6 cases of moderate to severe pain with partial response to analgesic therapy and 1 case (2%) of thrombosis of a residual haemorrhoidal nodule, all regressing after conservative therapy (Table 1). No severe complications or significant bleeding were reported. Postoperative complications were not statistically significantly associated with the number of nodules treated (1, 2, or 3), the grade of disease (2nd vs. 3rd degree) or the anticoagulant treatment (Tables 2–4).

**Table 2:** Comparison of the complication rate according with the number of hemorrhoidal nodules treated during the procedure using the Fisher's exact test. A *p*-value < 0.05 was considered statistically significant.

	Number of treated nodules				Fisher exact probabily test (*α*** **=** **0.05) two-tailed
	1	2	3	Total	
No complications	17	2	25	44	
Complications	0	0	7	7	
Total patients (*n*)	17	2	32	51	***p = 0.115** (p* * > 0.05* *)*

**Table 3:** Comparison of the complication rate according with the HD Degree of the patients included in the study using the Fisher's exact test. A *p*-value < 0.05 was considered statistically significant.

	II vs. III degree patients			Fisher exact probabily test (*α**** ***=*** ***0.05) two-tailed
	Complications	No complications	Total	
**II degree group**	6	21	27	
**III degree group**	1	23	24	
Total	7	44	51	***p = 0.1034*** *(p > 0.05)*

**Table 4:** Comparison of the complication rate according with the anticoagulant/antiplatelet regimen of the patients included in the study using the Fisher's exact test. A *p*-value < 0.05 was considered statistically significant.

	Anticoagulant/antiplatelet therapy					
	Dual antiplatelet	Single antiplatelet	NOACS	Other	Total	Fisher exact probabily test (*α*** **=** **0.05) two-tailed
**No complications**	21	8	10	5	44	
**Complications**	5	1	1	0	7	
Total patients (*n*)	26	9	11	5	51	***p = 0.87*** *(p > 0.05)*

Only one patient (2%), who was taking a single antiplatelet medication (acetylsalicylic acid), required local anesthesia with 2% Mepivacaine (Carbocaine), and experienced an uneventful postoperative recovery.

During the follow-up, 2 patients (4%) required a new procedure for recurrent bleeding: one was treated with infrared photocoagulation on an outpatient basis, and the second required an open haemorrhoidectomy after 3 months. No cases of intraoperative or postoperative mortality occurred.

**Figure 5:**
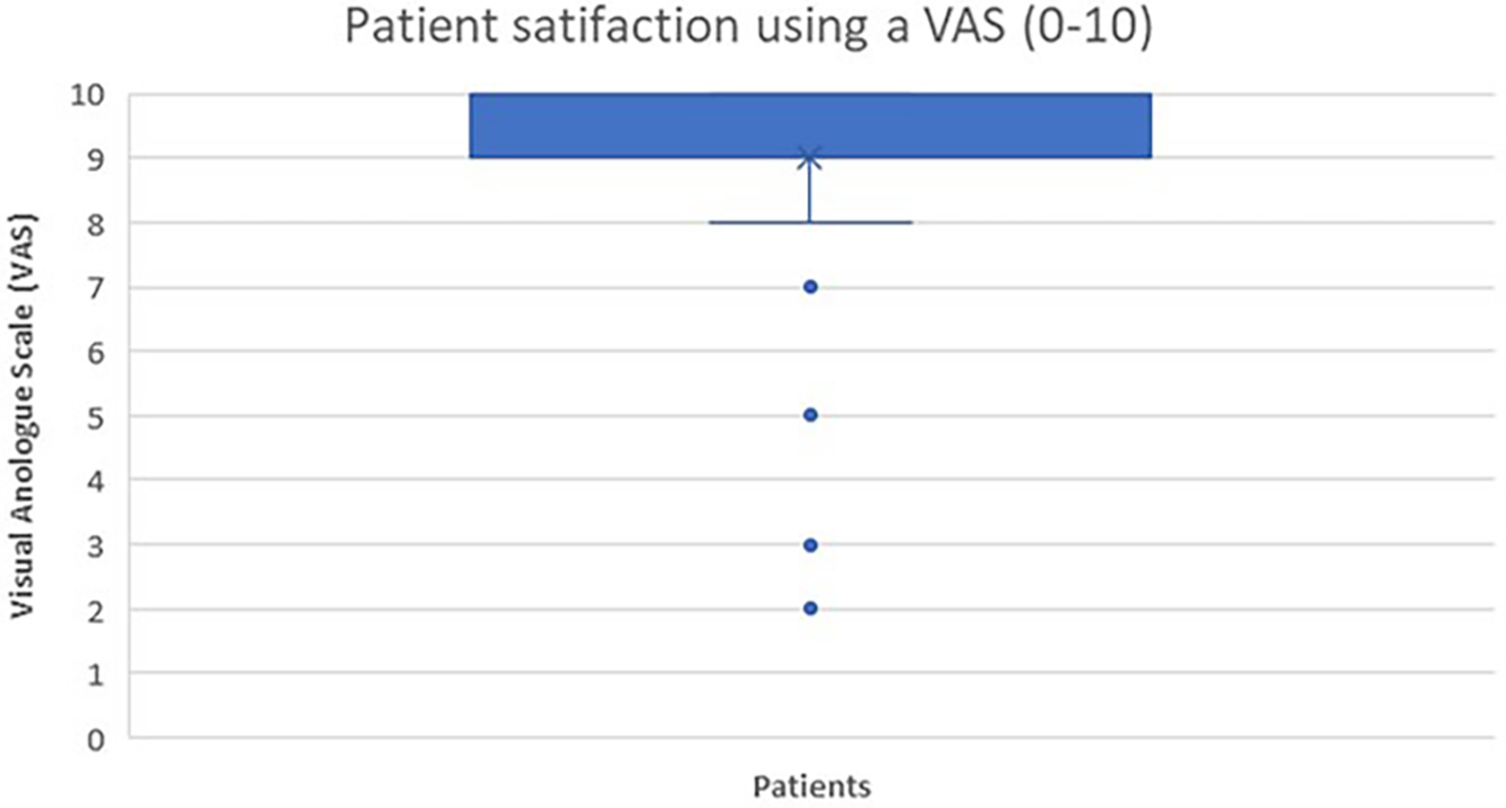
Box plot showing the patient satisfaction assessed by a VAS (visual analogue scale) 0-10.

Forty patients (78.43%) expressed a high level of satisfaction, rating 8–10 the procedure in a VAS scale (0-10) (Figure 5).

## Discussion

Our study shows that Sclerobanding is a feasible technique in patients on anticoagulant/antiplatelet treatment with a high safety profile, considering the absence of significant bleeding, intraoperative accidents, or severe postoperative complications. In addition to the feasibility and safety of the method, which were already demonstrated in a previous study on non-anticoagulated patients (20), it offers simplicity of execution, a short learning curve, cost-effectiveness (the procedure costs approximately 30 euros), repeatability in case of recurrence, and it usually does not require anaesthesia.

The complication rate was 13.7%, but it was represented only by 1 case of internal haemorrhoidal thrombosis [detected under sedation with an evaluation under anaesthesia (EUA) to reduce the patient's severe discomfort and treated conservatively], and 6 cases of moderate to severe pain, all regressed within 20 days. The absence of severe complications is the major advantage of the method.

The possibility of not discontinuing the anticoagulant treatment in the perioperative period contributes further to the safety profile, preventing the occurrence of serious thrombotic complications related to therapy suspension and rebound hypercoagulability, without an increased bleeding risk. This is particularly important since most experts suggest suspending anticoagulant or antiplatelet drugs at least one week before and 1–2 weeks after a surgical or an office-based procedure to reduce the risk of severe late bleeding, as described several times in the literature (21–23).

In an observational study of 1,269 proctologic interventions, the frequency of postoperative bleeding was 5%, with episodes occurring up to the 18th postoperative day, requiring transfusions and/or haemostasis in the operating theatre in a quarter of patients (24). The frequency rose to 33% in patients who had temporarily suspended NOAC therapy in favour of low-molecular-weight heparins. Considering that proctologic interventions require no or minimal hospitalization, the possibility of late bleeding events in these patients at home represents an additional risk. In particular, the use of clopidogrel seems to entail an additional risk compared to other anticoagulants. Nelson et al. (25), in a sample of 364 patients on anticoagulant or antiplatelet therapy undergoing rubber band ligation for haemorrhoids, reported 23 cases (3.7%) of postoperative bleeding, but only 6 severe cases (0.9%) requiring hospitalization or blood transfusion. However, 6 of these cases occurred in the 18 patients taking clopidogrel, 3 of which presented severe bleeding. Such data have been confirmed by other studies (23, 26). In our study, the use of clopidogrel alone or in dual antiplatelet therapy with acetylsalicylic acid (the largest group of patients) did not result in an increased risk of bleeding.

In a 4-year retrospective study, Martin et al. (27) investigated 111 patients necessitating hospitalization due to post-proctologic surgery bleeding. The study revealed significant associations between postoperative bleeding and factors such as hemorrhoid surgery, anticoagulant treatment (particularly direct oral anticoagulants), and an ASA score of 3. Notably, active smoking exhibited a protective effect, consistent with previous findings by other authors (28), likely attributed to smoking-induced vasoconstriction at the surgical site.

However, active smoking is a well-documented adverse factor on both wound healing and cardiovascular system, particularly in patients with other risk factors. Consequently, we recommended all treated patients to discontinue active smoking, although the compliance with this recommendation was not assessed.

Our study primarily included subjects on dual antiplatelet therapy and on NOAC therapy, at higher risk of postoperative bleeding, while most published studies focus on single antiplatelet therapy (acetylsalicylic acid and clopidogrel) or oral anticoagulant therapy (OAC).

Atallah et al. (1), in a retrospective study involving 106 subjects, reported 36 patients on anticoagulant/antiplatelet therapy, who underwent dearterialization with mucopexy (THD) without discontinuing ongoing therapy except for the day of the procedure. No statistically significant differences in postoperative complications were recorded, and postoperative bleeding events were similar between patients on anticoagulant therapy and healthy patients (19.4% vs. 15.7%, odds ratio 1.295, 95% CI 0.455–3.688, *p* = 0.785).

However, the study was limited by the heterogeneity of the anticoagulant drugs used, with the majority of patients (25 out of 36, or 69.4%) being solely on acetylsalicylic acid therapy, often at a low-dose regimen (81 mg/day), thereby posing challenges for generalizing these findings. The procedure also required regional or general anaesthesia, which can potentially lead to additional complications.

In another prospective study (29), including 73 patients with bleeding disorders who underwent sclerotherapy alone with 3% polidocanol foam, the complication rate was 9.6%, but with only 6 patients under dual antiplatelet therapy and 31 patients (42.4%) requiring a second or third session to treat the bleeding in a 1-year follow-up.

Regarding the grade of disease, the type of anticoagulant treatment and the number of nodules treated, no statistically significant differences were detected between subgroups. However, all complications occurred in the subgroup with 3 nodules treated during the same procedure. Thus, the lack of statistical significance might be attributed to the small sample size.

During the pandemic and in the post-pandemic period, the ability to treat the majority of patients with haemorrhoidal disease on an outpatient basis offers several advantages. This approach helps to preserve healthcare resources, such as hospital beds, operating rooms, and anaesthesiologists, which can then be directed toward critical care needs (30, 31). It also contributes to the reduction of waiting lists for other surgical conditions (20). Moreover, this outpatient approach can have a substantial impact on cost savings, the organization of the healthcare system, and the overall well-being of the patient. Patients benefit from avoiding hospital admissions and the associated risk of severe nosocomial infections (32).

In addition, the positive assessment provided by the patients further underscores the feasibility and the high acceptance rate of the technique.

The present study has inherent limitations due to its design and the small sample size. However, the sample size reflects a highly selected group of patients. We did not conduct any symptom evaluation scoring before and after the procedure. Nevertheless, the primary objective of the study was to assess the safety of the procedure and the absence of major complications, as outlined in the methods section. The procedure was primarily indicated to control recurrent bleeding, which could have had catastrophic consequences for this group of patients, rather than specifically addressing other symptoms. Therefore, the consideration for a new treatment during the follow-up period was linked to suboptimal results in controlling recurrent bleeding and the potential effects thereof.

The safety and feasibility results obtained in a highly selected group of high-risk patients and the potential benefits of the systematic use of the method represent a starting point for planning further multicentre studies with larger samples and longer follow-ups, necessary to confirm these promising preliminary results.

## Conclusions

Sclerobanding is a safe and effective technique in treating intermediate-grade haemorrhoidal disease (2nd and 3rd degree according to Goligher) in patients at high risk on anticoagulant/antiplatelet therapy. Sclerobanding is repeatable, usually does not require anaesthesia, and is cost-effective. Observational multicentre studies with a larger number of patients and controlled clinical trials will be needed to confirm these results.

## Data Availability

The raw data supporting the conclusions of this article will be made available by the authors, without undue reservation.
